# Implantable Cardioverter-Defibrillators for Secondary Prevention in Giant Cell Myocarditis

**DOI:** 10.7759/cureus.82391

**Published:** 2025-04-16

**Authors:** Krunal Shukla, Eric J Basile, Tawfiq Khasawneh, Mason Lin, Stephen J Brand

**Affiliations:** 1 Department of Internal Medicine, University of Florida College of Medicine, Gainesville, USA

**Keywords:** giant cell myocarditis, icd, im, internal medicine, medicine, ventricular tachycardia

## Abstract

Giant cell myocarditis (GCM) presents significant challenges in clinical management, particularly regarding the role of implantable cardioverter-defibrillators (ICDs) for secondary prevention of ventricular tachycardia (VT). We present the case of a 42-year-old female patient with histologically confirmed GCM who underwent orthotopic heart transplantation and subsequently developed VT due to biopsy-proven recurrent GCM within one year after transplant. An ICD was placed for secondary prevention following multiple episodes of monomorphic nonsustained VT. The patient would then present to the hospital for an ICD shock with device interrogation showing one episode of sustained VT. On further work-up, the patient was found to have a biopsy-proven recurrence of GCM. In this context, we review the existing literature, primarily case reports, small case series, and registry data, pertaining to ICD use in GCM. Our case underscores the lack of standardized guidelines and limited high-quality data supporting ICD placement in this population. It highlights the importance of individualized decision-making and contributes to clinical discussions by emphasizing the potential role of ICDs in managing life-threatening arrhythmias associated with both native and recurrent GCM.

## Introduction

Giant cell myocarditis (GCM) is an uncommon but highly aggressive autoimmune inflammatory disorder of the myocardium, characterized histologically by a T-cell-mediated infiltrate and multinucleated giant cells. The disease primarily affects young and middle-aged adults and is associated with a one-year mortality rate approaching 70%, underscoring its fulminant and often lethal course. Clinical deterioration is frequently rapid, with progression to advanced heart failure, malignant ventricular arrhythmias, and sudden cardiac death [1].

Among the most challenging aspects of GCM management is the prevention of fatal arrhythmias, including ventricular tachycardia (VT) and ventricular fibrillation. While implantable cardioverter-defibrillators (ICDs) are widely accepted for secondary prevention in many cardiomyopathies, their role, particularly in the context of transplantation or disease recurrence, remains ill-defined. The lack of formal guidelines and limited data complicate decision-making regarding prophylactic ICD placement in this high-risk population.

The clinical manifestations of GCM are heterogeneous and frequently overlap with other cardiac pathologies, rendering timely diagnosis a persistent challenge. Accurate identification relies on a combination of advanced cardiac imaging modalities and histopathologic confirmation by endomyocardial biopsy. Immunosuppressive therapy, typically involving high-dose corticosteroids and additional immunosuppressive agents, constitutes the mainstay of treatment. Long-term immunosuppression is generally required to sustain remission and prevent relapse [1].

In cases of refractory or fulminant disease, advanced therapies, including mechanical circulatory support, heart failure-directed pharmacologic management, and orthotopic heart transplantation, are often essential. Recurrence of GCM in the allograft, though uncommon, has been reported in approximately 20%-25% of cases and may present with conduction abnormalities or ventricular arrhythmias [2]. While most recurrences respond favorably to intensified immunosuppression, they still pose a significant risk for morbidity and mortality, highlighting the need for vigilant posttransplant surveillance and consideration of arrhythmia prophylaxis [2,3].

We report a rare case of recurrent GCM complicated by sustained VT in a heart transplant recipient managed with a shock from her cardiac device. This case contributes to the evolving discussion around ICD use in GCM and emphasizes the current lack of consensus regarding secondary prevention strategies in patients with GCM, as well as transplant recipients with recurrent disease.

## Case presentation

A 25-year-old female patient with a known history of GCM, ICD implantation, and status post-orthotopic heart transplantation presented to the emergency department after experiencing an ICD shock at home. Her posttransplant course had been complicated by left external iliac and common femoral artery occlusion, valganciclovir-resistant cytomegalovirus (CMV) viremia with colitis, and biopsy-proven cellular rejection within the first posttransplant year. Several months before this presentation, the patient had reported palpitations and was found to have episodes of nonsustained ventricular tachycardia (NSVT) on ambulatory monitoring. In light of her history of GCM and the emergence of ventricular arrhythmias, a shared decision was made with her electrophysiology team to implant an ICD for secondary prophylaxis.

**Figure 1:**
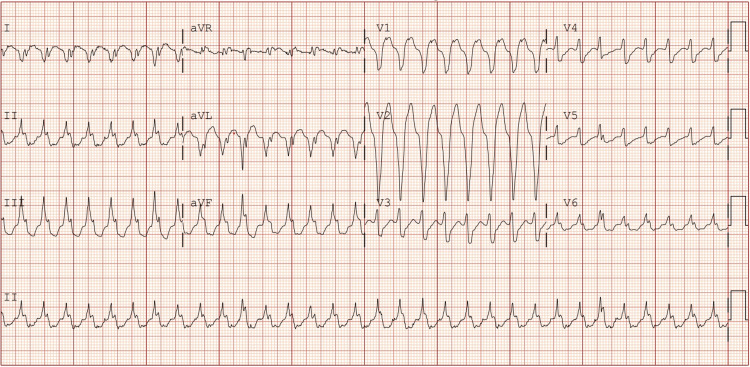
EKG consistent with monomorphic ventricular tachycardia aVR: augmented vector right; aVL: augmented vector left; aVF: augmented vector foot

**Figure 2:**
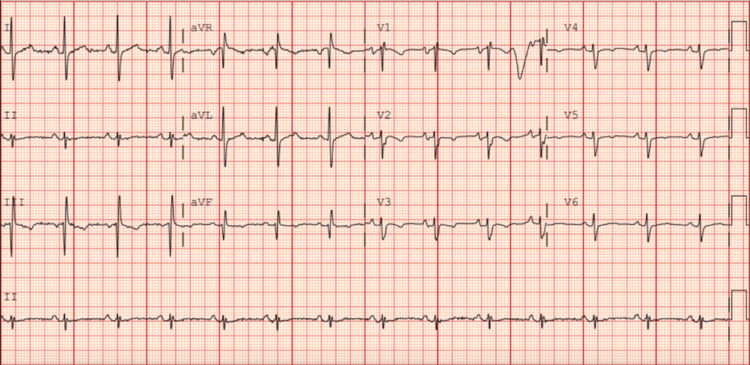
EKG demonstrating normal sinus rhythm with narrow QRS complexes and t-wave inversions in leads III, V1, V2, and V3 aVR: augmented vector right; aVL: augmented vector left; aVF: augmented vector foot

At the current presentation, the patient presented with a shock from her cardiac device. She was hemodynamically stable with normal vital signs. Serum studies were unremarkable except for mild, asymptomatic anemia (Table 1). Device interrogation revealed one episode of sustained monomorphic VT at a rate of 182 beats/minute, lasting 38 seconds, which triggered antitachycardia pacing followed by a single shock that restored sinus rhythm. Electrocardiography at presentation confirmed a wide-complex monomorphic VT with a right bundle branch block morphology and superior axis (Figure 1). There was no syncope, but the patient reported presyncope and palpitations before the shock. The patient was admitted for telemetry monitoring and initiated on a loading dose of intravenous amiodarone, followed by a maintenance infusion. This decision was based on her prior arrhythmia history, immunosuppressive regimen, and the relatively favorable safety profile of amiodarone in posttransplant patients. Alternatives such as sotalol and lidocaine were considered, but amiodarone was favored for its efficacy in sustained VT and low risk of proarrhythmia in the transplant population. She chemically converted to sinus rhythm shortly thereafter (Figure 2) and remained stable without recurrence during the hospitalization.

**Table 1:** Initial admission laboratory test results with reference ranges

Laboratory test result	Result	Units	Reference range
Sodium	139	mmol/L	135-145
Potassium	4.2	mmol/L	3.5-5
Chloride	106	mmol/L	98-107
Carbon dioxide	22	mmol/L	22-29
Urea nitrogen	17	mg/dL	7-20
Creatinine	1.16	mg/dL	0.74-1.35 (men), 0.59-1.04 (women)
White blood cells	13.1	×10³/µL	4-11
Hemoglobin	11.2	g/dL	13.5-17.5 (men), 12-15.5 (women)
Platelets	199	×10³/µL	150-450

Given the concern for disease recurrence, an endomyocardial biopsy was performed. Histopathology revealed a dense inflammatory infiltrate with a predominance of lymphocytes, areas of myocyte necrosis, and scattered multinucleated giant cells, findings consistent with recurrent GCM. The current sample demonstrated more diffuse inflammation and increased myocyte damage compared to prior biopsy specimens. The in-house cardiac transplant team recommended intensification of her immunosuppression, specifically by increasing the dose of mycophenolate mofetil while continuing baseline tacrolimus and prednisone. Due to the diffuse myocardial involvement and potential risk of worsening conduction system disease, catheter ablation was deemed inappropriate in this case.

After 48 hours without further arrhythmias on telemetry, the patient was discharged with close outpatient follow-up arranged through the heart transplant and electrophysiology teams. She was continued on oral amiodarone and her revised immunosuppressive regimen. Ongoing outpatient management includes scheduled surveillance endomyocardial biopsies that have yet to occur, serial imaging with cardiac MRI whose frequency will be determined by the outpatient electrophysiology service, and continued ICD monitoring/setting adjustments. Plans for long-term management include reassessment of arrhythmia burden, tapering of amiodarone based on stability, and further adjustments to immunosuppression based on biopsy findings and tolerance.

## Discussion

Effectively managing VT in GCM remains a significant clinical challenge due to the rarity of the condition and the lack of robust, evidence-based guidelines. In this case report and selective literature review, drawing primarily from case reports and observational studies, we explore the role of ICDs for secondary prevention in patients with GCM, particularly those who have undergone heart transplantation.

Our patient developed nonsustained VT several months posttransplant, leading to ICD implantation. While this scenario aligns more closely with secondary prevention, given prior documented ventricular arrhythmia, it highlights a unique clinical gray zone. NSVT in transplant recipients with known GCM recurrence may suggest an evolving arrhythmic substrate, though it does not meet the traditional threshold for secondary prevention as defined by sustained VT or cardiac arrest. This nuance further underscores the current gap in guidelines and the need for individualized assessment.

Based on our review of the literature, there are only a few reported cases of recurrent GCM in patients who received heart transplants for the condition [2]. There is a dearth of high-quality studies and the conspicuous absence of formal guidelines regarding ICD placement in GCM, highlighting the urgent need for further research to guide clinical decision-making in this specific domain. Our case adds to this limited literature, representing a rare instance of biopsy-confirmed GCM recurrence complicated by sustained VT managed with ICD therapy. Unlike most prior cases, this patient had multiple transplant-related complications, including CMV viremia, prior rejection, and complex vascular disease, which may have influenced both immune function and arrhythmia risk. The presence of irreversible substrate and triggers of ventricular arrhythmias, including GCM, supports the use of ICD [3]. While ICD placement may confer benefits in select cases, discerning its appropriateness necessitates meticulous evaluation on an individual basis, weighing potential risks and benefits. However, we found no documented cases of VT managed with prophylactic ICDs in patients with heart transplants for GCM. Given the potential for recurrence up to nine years after transplant, patients with a history of GCM should have lifelong, frequent follow-ups, ideally every three to six months [4].

Guidelines from professional societies such as the Heart Rhythm Society and the International Society for Heart and Lung Transplantation currently recommend ICD implantation in transplant recipients who experience sustained or hemodynamically significant ventricular arrhythmias [5]. However, there are no established guidelines regarding the use of prophylactic ICDs in patients who have undergone heart transplantation due to GCM. Evidence from randomized trials supports primary prevention ICD implantation for patients with reduced left ventricular function after myocardial infarction or symptomatic heart failure, demonstrating a clear mortality benefit. This benefit is largely due to the ICD’s ability to terminate malignant VT or ventricular fibrillation, preventing sudden cardiac death [1,4]. Additionally, the studies by Chiu et al. and Ekström et al. underscore the association between GCM and life-threatening ventricular arrhythmias such as VT, further emphasizing the need for effective management strategies, including ICD placement [6,7]. These findings collectively underscore the critical importance of tailored approaches and ongoing research efforts in managing GCM-related VT. Catheter ablation was not pursued in our patient due to extensive, diffuse myocardial inflammation on biopsy and concern for worsening conduction abnormalities, considerations common in GCM and especially relevant in posttransplant recipients. Ablation remains an evolving area of research in GCM-related VT, with most data limited to highly selected patients with localized arrhythmogenic foci [8].

Ultimately, this case reinforces the importance of frequent, lifelong follow-up in patients with a history of GCM, including surveillance imaging, biopsy, and device monitoring. Given the risk of late recurrence, even years after transplant, clinicians must remain vigilant [4]. Our findings suggest that recurrent GCM with VT posttransplant represents a distinct clinical phenotype that may warrant earlier consideration of ICD therapy and closer immunologic monitoring. To advance care in this rare but high-risk population, multicenter registries or prospective cohort studies are needed to clarify the arrhythmic risk associated with GCM recurrence and better define ICD candidacy, particularly in transplant recipients. Standardized protocols for follow-up intervals, arrhythmia surveillance, and immunosuppressive modulation could also improve outcomes and reduce variability in care.

## Conclusions

This case underscores the exceptional rarity of recurrent GCM in heart transplant recipients and illustrates how an ICD shock served as the sentinel event leading to the diagnosis of recurrence. It supports consideration of ICD use for secondary prevention in transplant patients with a history of GCM who develop documented ventricular arrhythmias while also emphasizing the ongoing uncertainty due to the absence of specific guidelines for this population.

Given the potential for late GCM recurrence, even several years after transplant, and the unpredictable arrhythmic profile associated with the disease, a personalized approach should include individualized ICD programming (e.g., VT detection thresholds and therapy zones), regular endomyocardial biopsy surveillance, and tailored immunosuppression adjustments based on evolving risk. Multidisciplinary collaboration involving transplant cardiology, electrophysiology, heart failure, and pathology teams is essential to ensure comprehensive assessment and timely intervention. Long-term, frequent follow-up-potentially every three to six months-is critical to monitor for recurrence and arrhythmic complications. This case highlights the urgent need for prospective registries, consensus statements, and multicenter collaborations to define optimal management strategies, clarify ICD indications, and improve outcomes for this high-risk and understudied patient population.
